# Percutaneous Sclerotherapy of Venous Malformations of the Hand: A Multicenter Analysis

**DOI:** 10.1007/s00270-021-02926-x

**Published:** 2021-07-20

**Authors:** Vanessa F. Schmidt, Max Masthoff, Constantin Goldann, Sinan Deniz, Osman Öcal, Beate Häberle, Michael Köhler, Max Seidensticker, Jens Ricke, Walter A. Wohlgemuth, Richard Brill, Moritz Wildgruber

**Affiliations:** 1 Department of Radiology, University Hospital, LMU Munich, Marchioninistr 15, 81377 München, Germany; 2 Clinic of Radiology, University Hospital Muenster, Muenster, Germany; 3 Clinic and Policlinic of Diagnostic Radiology, Martin-Luther University Halle-Wittenberg, Halle (Saale), Germany; 4 Department for Pediatric Surgery, University Hospital, LMU Munich, München, Germany

**Keywords:** Venous malformation, Upper extremity, Hand, Sclerotherapy, Interventional radiology

## Abstract

**Purpose:**

To evaluate the safety and outcome of percutaneous sclerotherapy for treating venous malformations (VMs) of the hand.

**Materials and Methods:**

A retrospective multicenter trial of 29 patients with VMs primarily affecting the hand, including wrist, carpus, and/or fingers, treated by 81 percutaneous image-guided sclerotherapies using ethanol gel and/or polidocanol was performed. Clinical and imaging findings were assessed to evaluate clinical response, lesion size reduction, and complication rates. Substratification analysis was performed with respect to the Puig’s classification, the sclerosing agent, the injected volume of the sclerosant, and to previously performed treatments.

**Results:**

The mean number of procedures per patient was 2.8 (± 2.2). Last follow-up (mean = 9.2 months) revealed a partial relief of symptoms in 78.9% (15/19), while three patients (15.8%) presented symptom-free and one patient (5.3%) with no improvement. Post-treatment imaging revealed an overall objective response rate of 88.9%. Early post-procedural complications occurred after 5/81 sclerotherapies (6.2%) and were entirely resolved by conservative means. Type of VM (Puig’s classification) as well as sclerosing agent had no impact on clinical response (*p* = 0.85, *p* = 0.11) or complication rates (*p* = 0.66, *p* = 0.69). The complication rates were not associated with the sclerosant volume injected (*p* = 0.76). In addition, no significant differences in clinical success (*p* = 0.11) or complication rates (*p* = 0.89) were detected when comparing patients with history of previous treatments compared to therapy-naive patients.

**Conclusion:**

Percutaneous sclerotherapy is both safe and effective for treating VMs of the hand. Even patients with history of previous treatments benefit from further sclerotherapy showing similar low complication rates to therapy-naive patients.

**Level of Evidence:**

Level 4, Retrospective study.

## Introduction

Venous malformations (VMs) are the most common congenital vascular malformations and occur with a prevalence of up to 1% in the overall population [1, 2]. Due to errors in endothelial cell morphogenesis and disorganized vasculogenesis, these lesions show a dilated, dysplastic, and hemodynamically nonfunctional venous-like network [3, 4]. In many cases, venous malformations grow proportionally during childhood and may remain unnoticed for years prior to symptomatic clinical presentation. As clinical relapse after surgical resection of vascular malformations is generally frequent [5] and surgical options may be challenging or limited when VMs are located at the hand or wrist, percutaneous sclerotherapy using various sclerosants such as gelified ethanol or polidocanol (liquid or foam) has evolved as a minimally invasive alternative [1, 4, 6, 7]. Patients’ symptoms leading to clinical presentation and treatment vary with extent and localization of the VM and include swelling, recurrent or chronic pain, motor or sensory impairment, inflammation and ulceration as well as an increased risk of thromboembolic symptoms [8]. Therefore, the treatment of VMs in small anatomical compartments such as hand and wrist with dense innervation and small functional units is potentially associated with increased risk of adverse events. Additionally, blood flow in the terminal vasculature of the fingers may easily be compromised. Consequently, higher rates of post-procedural muscle contracture, nerve injury, impaired mobility, and compartment syndrome have been reported for this location [2, 9]. The purpose of this multicenter study was to evaluate the safety and clinical outcome of percutaneous sclerotherapy for the treatment of VMs affecting the hand, including wrist, carpus and/or fingers.

## Materials and Methods

This multicenter study was approved by the local ethics committee (University Hospital, LMU Munich, protocol No.: 21–0264) and was performed in accordance with relevant guidelines and regulations according to the Helsinki Declaration of 2013. Patients were recruited via the interdisciplinary Vascular Anomalies Centers at three tertiary care university hospitals.

**Fig. 1 A:**
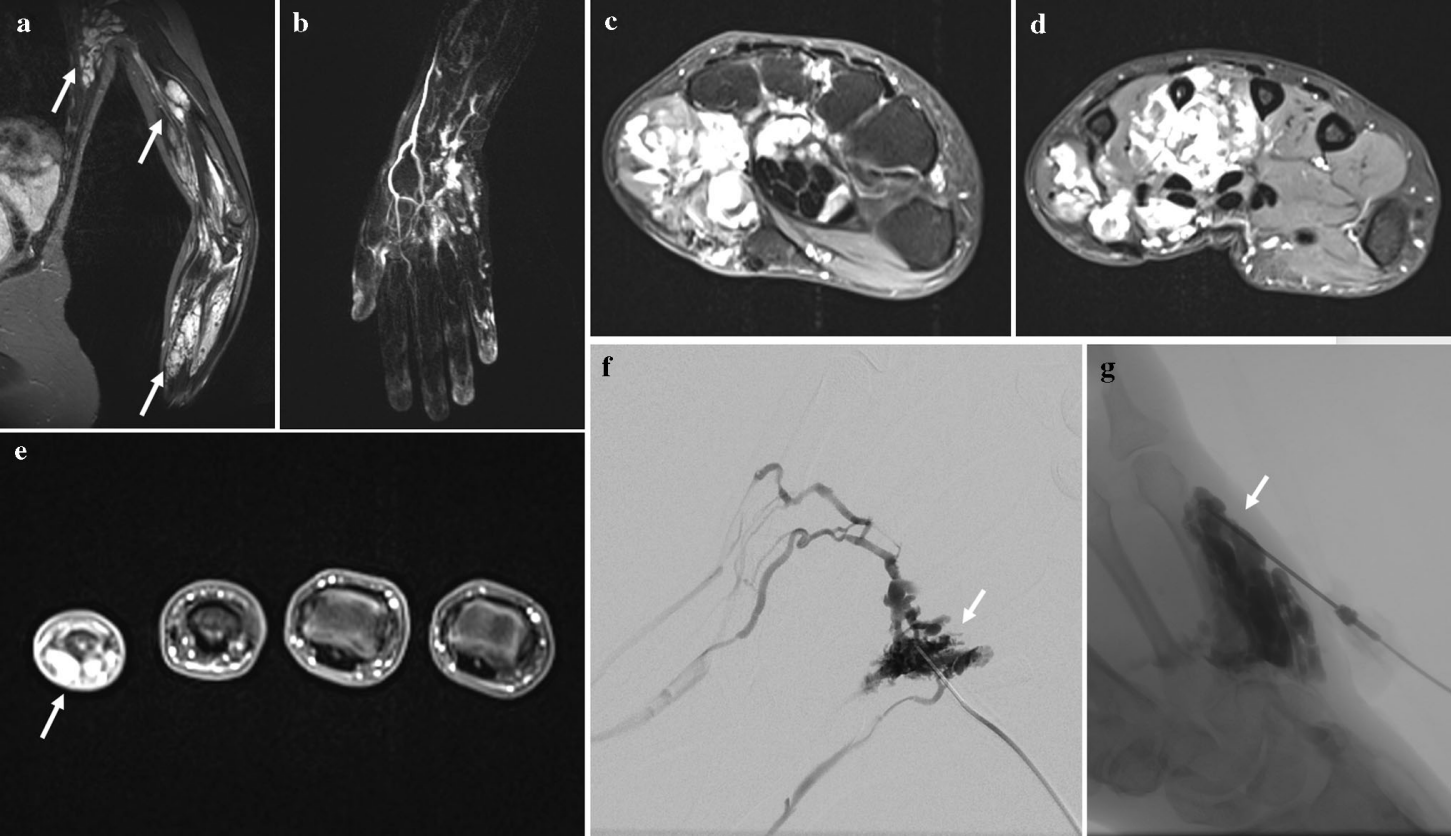
29-year-old female patient with extensive findings of VMs in form of multifocal lesions affecting the left upper extremity. **a** Coronar T1-weighted fat-saturated fast spin-echo MR image demonstrates three separated hyperintense masses of tubular structures (arrows) extending into the flexor muscles of forearm and humerus and the latissimus dorsi muscle. **b**-**d** Time-resolved 3D MR angiography and T1-weighted fat-saturated contrast-enhanced MR images show the inferior venous malformation with extension from the middle of the forearm up to the metacarpalia. **e** T1-weighted fat-saturated contrast-enhanced MR image reveals involvement of the fifth finger (arrow). **f** + **g** Negative roadmap images demonstrate drainage of a lesion (arrow) in normal veins corresponding to type II (Puig’s classification) and filling of another lesion within the wrist and the hypothenar region of the right hand (arrow) without venous drainage

A total of 29 consecutive patients, 10 males and 19 females, with VMs of the hand treated with 81 percutaneous sclerotherapies between 2017 and 2021 were analyzed retrospectively. Patient characteristics are summarized in Table 1. The VMs were diagnosed during the clinical presentation by a combination of physical examination and imaging including magnetic resonance imaging (MRI) and ultrasound. All patients suffered from VMs affecting primarily the hand, including wrist (18/29, 62.1%), carpus (22/29, 75.9%), and/or fingers (16/29, 55.2%). Six of 29 patients (20.7%) presented with multifocal manifestation (see Fig. 1), while none showed VM associated with other anomalies (such as Klippel–Trenaunay syndrome) or combined vascular malformations. The indications for percutaneous sclerotherapy were pain, swelling, cosmetic disfigurement, or functional impairment. Both therapy-naive patients (15/29, 51.8%) and patients having undergone previous invasive treatments (14/29, 48.2%) by debulking surgery (9/29, 31%), sclerotherapy (2/29, 6.9%), or both (3/29, 10.3%) without sufficient symptom improvement were included. The Puig’s classification of lesions was performed on pre-interventional MRI scans and is included in Table 1.

**Table 1:** Patient and clinical characteristics of study cohort (*n* = 29)

Characteristic		Total cohort (*n* = 29)
Age at diagnosis	Mean (standard deviation)	16.3 (± 16.7)
Age at treatment initiation	Mean (standard deviation)	23.2 (± 19.3)
Men		10 (34. 5%)
*Location side*		
Right		16 (55.2%)
Left		13 (44.8%)
*Appearance*		
Multifocal		23 (79.3%)
Isolated		6 (20.7%)
*Puig’s classification*		
Type 1		18 (62.1%)
Type 2		5 (72.2%)
Type 3		4 (13.8%)
Type 4		2 (6.9%)
*Involved anatomical structures*		
Wrist		18 (62.1%)
Carpus		22 (75.9%)
Finger		16 (55.2%)
*Treatment rationales*		
Pain		24 (82.8%)
Swelling		21 (72.4%)
Functional impairment		18 (62.1%)
Cosmetic disfigurement		8 (27.6%)
*Number of procedures*		
1		10 (34.5%)
2		9 (31.0%)
3		3 (10.3%)
4		1 (3.4%)
5		1 (3.4%)
6		3 (10.3%)
7		1 (3.4%)
8		0 (0.0%)
9		0 (0.0%)
10		1 (3.4%)

Interventional treatment was carried out under general anesthesia. Postoperative medication consisted of ibuprofen 10 mg/kg/KG per day and weight-adapted low-molecular weight heparin in a prophylactic dose for seven days. Sclerotherapy was performed under real-time ultrasound and fluoroscopic guidance using gelified ethanol (Sclerogel®, ab medica GmbH & Co. KG, Düsseldorf, Germany; Discogel®, 1A Medical AG, Hettlingen, Schweiz) and/or 2–3% polidocanol foam (Aethoxysclerol®, Kreussler & Co. GmbH, Wiesbaden, Germany; ratio of polidocanol to sterile air was 1:4). Gelified alcohol was used in case of rapid venous drainage toward larger draining veins. A maximum of 6 mL gelified ethanol and 10 mL of polidocanol foam were used per session. Repetitive sclerotherapy procedures were performed depending on the extent of the lesion, response to therapy, and course of clinical symptomatology. The patients were seen within a standardized follow-up regime in the three centers involved. The first clinical follow-up was performed at 1–3 months after each sclerotherapy session. In case of no additional treatment, the first follow-up MRI was scheduled at 6 months. Additional clinical follow-up and MRI were conducted at 12 and 24 months.

Retrospective data collection was performed using electronic patient records and the picture archiving and communication system (PACS) at each department. Data analysis was conducted centrally to evaluate patient/demographic data and to define Puig’s classification, clinical success, objective outcome (imaging), and complication rates. Clinical success at follow-up was measured using the following grading scale: symptom-free, partial relief of symptoms, no improvement of symptoms, and clinical progression under sclerotherapy. Objective outcome was assessed by changes in VM size using pre- and post-procedural MRI for subdivision of image findings into the following four categories: complete response (CR, 100% VM size reduction), partial response (PR, ≥ 30% VM size reduction), stable disease (SD, neither PR nor PD criteria met), progressive disease (PD, ≥ 20% VM size increase). Lesion size was assessed on delayed-phase contrast-enhanced fat-saturated T1-weighted images using the largest lesion diameter in one imaging plane, comparable to the response evaluation criteria in solid tumors (RECIST). Complications were classified as early complications occurring within the first 30 days after the intervention and late complications arising thereafter.

Substratification analyses were performed depending on Puig’s classification [10], the type of sclerosant, the injected volume of the sclerosant, and with respect to previous treatment (surgery and/or minimally invasive therapy) versus therapy-naive patients. For analysis, the Pearson’s Chi-squared test was used for categorial data and the Mann–Whitney U test for metric data. Statistical testing was conducted using SPSS (version 26.0, IBM Corp., Armonk, NY, USA), with *p* < 0.05 considered significant.

## Results

The median follow-up period after the last sclerotherapy session was 6 months (range 1–26; mean 9.2 ± 9.1 months).

### Clinical Response

The mean number of percutaneous sclerotherapies per patient was 2.8 (± 2.2). The distribution of number of procedures per patient is shown in Table 1. A total of 62/81 sclerotherapies (76.5%) were performed using polidocanol foam with a mean injected volume of 4.4 mL (± 2.5). Of 81 treatments, nine (11.1%) were performed using gelified ethanol with a mean injected volume of 4.0 mL (± 2.3), and 10/81 procedures (12.3%) were performed using a combination of polidocanol foam and gelified ethanol. After the first sclerotherapy session, post-treatment follow-up was available in 23/29 patients (79.3%): 21 patients (91.3%) showed partial relief of symptoms, while two patients (8.7%) presented with no improvement. After the second sclerotherapy session, post-treatment follow-up was completely reported in 16/19 patients (84.2%): 15/16 patients (93.8%) had partial relief, and one patient (6.3%) showed no improvement of symptoms. After the third–tenth sclerotherapy sessions, post-treatment follow-up was available in 69.7%: Hereby, all patients showed partial relief of symptoms. A terminal follow-up after the last sclerotherapy was documented in 19/29 patients with partial relief of symptoms in 15/19 patients (78.9%), while 3/19 patients (15.8%) presented as symptom-free and one patient (5.3%) with no clinical improvement.

### Imaging Outcome

**Fig. 2:**
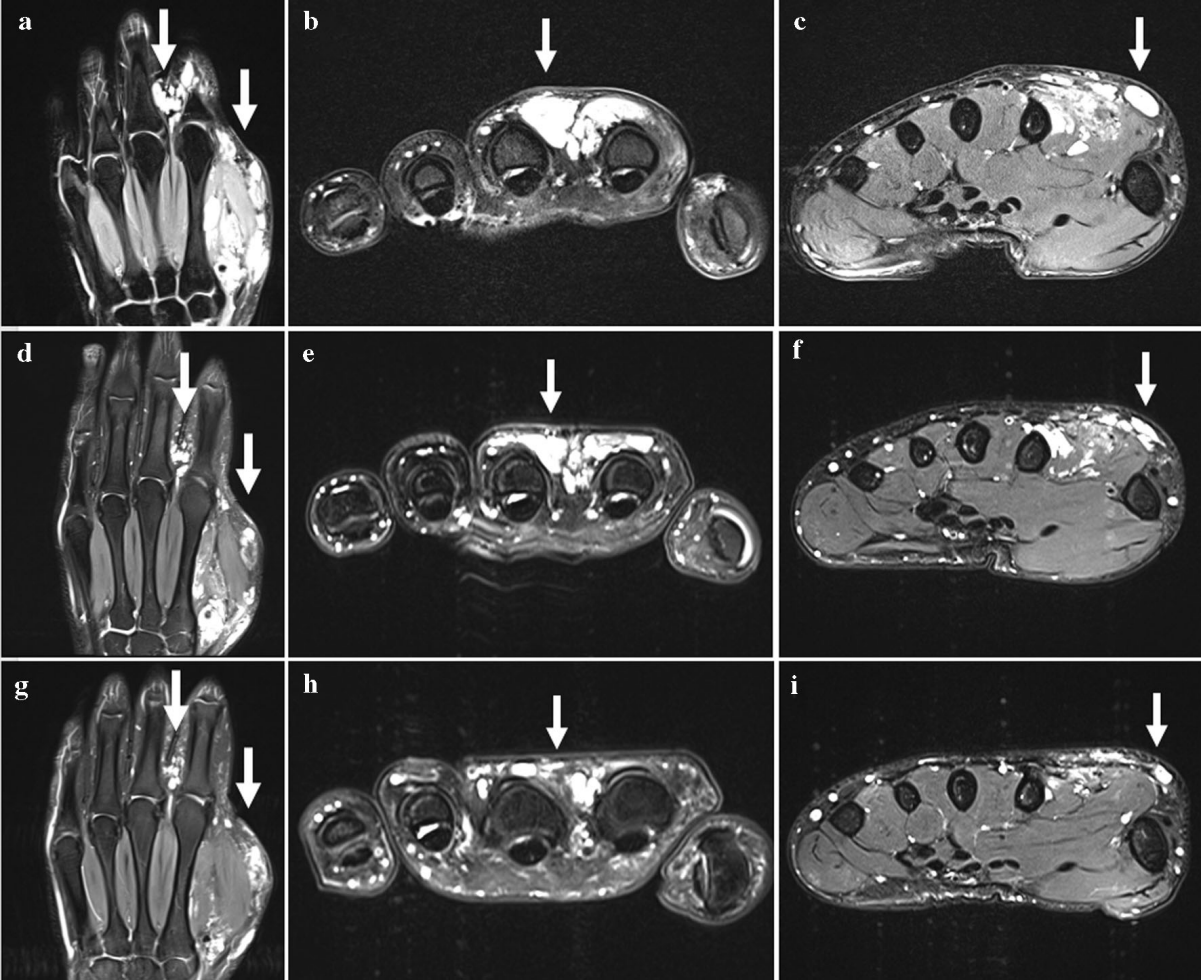
T1-weighted fast spin-echo fat-saturated MR images showing the hyperintense VM of a 23-year-old male patient at intervals between several sclerotherapy sessions. **a**-**c** Coronar and axial MR images demonstrate hyperintense tubular structures involving thenar and interdigital region of the left hand extending out to the dermis (arrows). **d**-**f** 4 months after treatment initiation: coronar and axial MR images reveal lesion size reduction (arrows) when compared to (**a**-**c**). **g**-**i** 9 months after treatment initiation: coronar and axial MR images show further lesion size reduction up to 50% (arrows) when compared to (**a**-**c**) and (**d**-**f**)

After the final sclerotherapy session, post-treatment imaging was available in 18/29 patients at terminal follow-up. Here, the changes in lesion size revealed PR in 15 patients (83.3%), SD in one patient (5.6%), CR in one patient (5.6%), and PD in one patient (5.6%), resulting in an overall objective response rate of 88.9% (16/18), see Fig. 2.

### Safety

Early complications (< 30 days) occurred after 5/81 sclerotherapies (6.2%) including thrombophlebitis beyond the treated area of malformation (2/81, 2.5%), prolonged swelling at injection side (> 7 days) (2/81, 2.5%), and hematoma at injection side (1/81, 1.2%). In all cases, complications were self-limited and entirely resolved with conservative means. No patient developed skin injury or soft tissue necrosis, local infection at injection site, sensory loss, or peripheral ischemia/necrosis/ulcer. Overall, no late complications were reported.

### Impact of Lesion Type and Procedural Characteristics

The type of VM according to the Puig’s classification showed neither a significant influence on the post-procedural complication rate (Chi-squared test, *p* = 0.66) nor on the clinical response at final follow-up (Chi-squared test, *p* = 0.85). In addition, there were no significant differences between the procedures performed with gelified ethanol compared to polidocanol foam regarding the incidence of post-procedural complications (Chi-squared test, *p* = 0.69) or the clinical response at terminal follow-up (Chi-squared test, *p* = 0.11). No significant differences between the sclerosant volume injected and the post-procedural complication rates were found (Mann–Whitney U test *p* = 0.76). Previous treatment (surgery or minimally invasive therapy) had no impact on clinical response or complication rates when compared to therapy-naive patients (Chi-squared test, *p* = 0.11, *p* = 0.89).

## Discussion

Several concerns for the use of percutaneous sclerotherapy in locations as wrist, carpus, or finger have been raised: Exemplarily, due to the risk of necrosis and compartment syndrome, Mendonca et al. [11] suggested, in the Birmingham experience, that sclerotherapy should not be considered for VMs in distal extremities. In contrast, Delgado et al. [2] reported an experience with 34 sclerotherapies for foot VMs in 16 patients with a significant improvement of clinical symptoms and a complication rate of 21%. In addition, Guevara et al. [9] published a series of 17 patients, who underwent 40 sclerotherapies of diffuse and infiltrative VMs of the distal forearm with a reported complication rate of 5%. Compared to the two latter studies, the present study reports a larger cohort consisting of 29 patients and an increased number of procedures with a total of 81 sclerotherapies. Depending on lesion size and clinical success, the mean number of procedures per patient was 2.8, which is comparable to 2.4 and 2.1 sclerotherapies per patient reported by Guevara et al. [9] and Delgado et al. [2]. With respect to the type and amount of sclerosant applied, the two latter studies used pure ethanol or STS foam, which cannot be directly compared to gelified alcohol and polidocanol. The amount of sclerosant in our study was rather low, which may account for the moderate complication rates. More aggressive approaches may be more effective but can potentially be accompanied with higher complication rates, which in our eyes should especially be considered in anatomically challenging locations such as the hand and wrist.

We could demonstrate that percutaneous sclerotherapy of VMs of the hand using gelified ethanol and/or polidocanol foam is effective with respect to clinical symptoms and imaging outcome at the three investigated Vascular Anomalies Centers. The terminal follow-up after the last sclerotherapy showed at least partial relief of symptoms in ~ 95% of patients. Our results are comparable to a smaller cohort with infiltrative and diffuse VMs of the hand published by Guevara et al. [9], in which partial relief was seen in the majority of patients (59%) and 24% of patients presented symptom-free. Guevara et al. [9] reported clinical progress in spite of sclerotherapy in 2%, which was not found in any patient of our cohort. Here, it should be mentioned that the collective of Guevara solely included complex cases of infiltrative and diffuse malformations, tending to respond worse to treatment, as reported by Ali et al. [12]. In our study, post-treatment imaging revealed an overall response rate of 88.9%, supported by the results of peripheral VMs at various locations published in the current literature. Exemplarily, Teusch et al. [13] reported an overall response rate of 93% in 31 prospective patients treated with gelified ethanol and Ali et al. [12] of 92% in 37 patients treated with polidocanol.

The present study showed a low complication rate of 6.2%, with all sequalae having resolved by conservative means. Guevara et al. [9] also reported a similarly low complication rate (5%) for the treatment of hand VM; however, in contrast to this cohort, these were all skin complications. In general, the most commonly described post-procedural complications of percutaneous sclerotherapy are skin blistering, ulceration, and/or necrosis, especially in superficial lesions [2, 13, 14]. As this type of complication resulting in potentially permanent impairment could be avoided in our study, the presented approach confirms the acceptable risk profile of both sclerosants used and makes them particularly attractive for repeated sclerotherapy sessions even in challenging anatomical locations.

Comparing gelified ethanol and polidocanol regarding the incidence of post-procedural complications or the clinical success at terminal follow-up, we found no significant differences in both subgroups in our series. Gelified ethanol, a composition of ethanol, supplemented with water-insoluble cellulose derivative and embedded by a cotton wool-like network, has numerous advantages in comparison with pure liquid alcohol. This includes longer contact time with the vessel wall, lower amount of ethanol needed per procedure, and consequently fewer systemic complications [4, 13]. Ierardi et al. [15] reported, in a small, retrospective cohort of six patients, post-treatment clinical success of 100% without any systemic side effects. Polidocanol is a frequently descripted detergent, causing lysis of vessel endothelium showing low complication rates [4, 12]. Grieb et al. [16] presented clinical success of 90% and a complication rate of 1.8% in 20 patients with craniofacial VMs. Compared to the current literature, our results confirm that both gelified ethanol and polidocanol are effective and safe sclerosants when used appropriately in treating VMs of the hand.

Though there is some evidence that higher sclerosant volumes may increase the incidence of post-procedural complications [6], we found no relation between the sclerosant volume and the complication rates. This is comparable to the results of Fayad et al. [17] and Delgado et. al. [2], which treated VMs by ethanol and/or sodium tetradecyl sulfate (STS) foam. This is due to the fact that besides the sclerosant volume, the number of puncture sites plays an important role. By using several access needles during sclerotherapy, it is possible to distribute the sclerosant agent over larger volumes, avoiding local peak concentrations at the injection sites.

The group of patients who had previously undergone surgical and/or minimally invasive treatments presented comparable clinical response at terminal follow-up with a similar low risk of complications as compared to therapy-naive patients. These results emphasize that even in the case of recurrence and insufficient symptom improvement after previous treatment, further sclerotherapy may be safe and effective, even if multiple sessions are necessary to achieve sustainable success.

Additionally, the Puig’s classification of VMs showed no significant influence on the rate of post-procedural complications or on the clinical success at terminal follow-up. Although it is reported that VMs categorized higher in Puig’s classification show less overall response as outflow veins maintain the consistency of the lesion, while the sclerosant is difficult to retain in the lesion without outflow vein occlusion [2, 10], we were able to achieve similar results with no differences in complication rates regarding the different Puig’s type of VM. An update of the classification published in 2002 may help to better differentiate between different subgroups of VM [18].

Limitations of the presented multicenter study include the retrospective design with the consecutive lack of standardized follow-up information for some patients. Clinical success was based on a simplified classification of symptom evolvement, rather than by a detailed evaluation of health-related quality of life. Similarly, no standardized numerical assessment of pain before and after treatment was available for a more objective assessment of the clinical burden. In addition, objective response post-treatment was measured by changes in VM size as assessed by MRI. For this purpose, no standardized protocol or recommendations/guidelines exist and the following established oncological mRECIST criteria may not be the best option for vascular malformations. In this regard, new functional imaging modalities may prove more versatile for diagnosis and treatment response evaluation in the future [19]. The mean follow-up time after the last sclerotherapy was 9.2 months representing a rather short period, in particular regarding the recurrence rate of vascular malformations, which are frequently recurring after a longer time period.

## Conclusion

This study reveals that sclerotherapy using gelified ethanol and/or polidocanol foam is both effective and safe for treating VMs of the hand. Repetitive procedures may be needed to achieve appropriate relief of symptoms and improved function. Previously surgically or minimally invasively treated patients similarly benefit from sclerotherapy while showing comparably low complication rates to therapy-naive patients.

## References

[1] Gorman J, Zbarsky SJ, Courtemanche RJM, Arneja JS, Heran MKS, Courtemanche DJ (2018). Image guided sclerotherapy for the treatment of venous malformations. CVIR Endovasc.

[2] Delgado J, Bedoya MA, Gaballah M, Low DW, Cahill AM (2014). Percutaneous sclerotherapy of foot venous malformations: evaluation of clinical response. Clin Radiol.

[3] Colletti G, Ierardi AM (2017). Understanding venous malformations of the head and neck: a comprehensive insight. Med Oncol.

[4] Hage AN, Chick JFB, Srinivasa RN (2018). Treatment of venous malformations: the data, where we are, and how it is done. Tech Vasc Interv Radiol.

[5] Lee BB, Baumgartner I, Berlien P (2015). Diagnosis and treatment of venous malformations consensus document of the international union of phlebology (IUP) updated 2013. Int Angiol.

[6] Rabe E, Pannier F (2013). Sclerotherapy in venous malformation. Phlebology.

[7] Hou F, Chen J, Xia M, Ding K, Zeng Q, Liu W (2020). Percutaneous sclerotherapy with polidocanol under the guidance of ultrasound for venous malformations in children - a retrospective cohort study from a single tertiary medical center. Medicine (Baltimore).

[8] Sierre S, Teplisky D, Lipsich J (2016). Vascular malformations: an update on imaging and management. Arch Argent Pediatr.

[9] Guevara CJ, Gonzalez-Araiza G, Kim SK, Sheybani E, Darcy MD (2016). Sclerotherapy of diffuse and infiltrative venous malformations of the hand and distal forearm. Cardiovasc Intervent Radiol.

[10] Puig S, Aref H, Chigot V, Bonin B, Brunelle F (2003). Classification of venous malformations in children and implications for sclerotherapy. Pediatr Radiol.

[11] Mendonca DA, McCafferty I, Nishikawa H, Lester R (2010). Venous malformations of the limbs: the Birmingham experience, comparisons and classification in children. J Plast Reconstr Aesthet Surg.

[12] Ali H, Saleh M, Mohammed W (2017). Efficacy and safety of Duplex-guided polidocanol foam sclerotherapy for venous malformations. Int Angiol.

[13] Teusch VI, Wohlgemuth WA, Hammer S (2017). Ethanol-gel sclerotherapy of venous malformations: effectiveness and safety. AJR Am J Roentgenol.

[14] Wohlgemuth WA, Müller-Wille R, Teusch V, Hammer S, Wildgruber M, Uller W (2017). Ethanolgel sclerotherapy of venous malformations improves health-related quality-of-life in adults and children - results of a prospective study. Eur Radiol.

[15] Ierardi AM, Colletti G, Biondetti P, Dessy M, Carrafiello G (2019). Percutaneous sclerotherapy with gelified ethanol of low-flow vascular malformations of the head and neck region: preliminary results. Diagn Interv Radiol.

[16] Grieb D, Meila D, Greling B (2019). Craniofacial venous malformations treated by percutaneous sclerotherapy using polidocanol: a single-center experience. Acta Radiol.

[17] Fayad LM, Hazirolan T, Carrino JA, Bluemke DA, Mitchell S (2008). Venous malformations: MR imaging features that predict skin burns after percutaneous alcohol embolization procedures. Skeletal Radiol.

[18] Müller-Wille R, Wohlgemuth WA (2018). Klassifikationen für venöse Malformationen – sind sie adäquat?. Gefässchirurgie.

[19] Masthoff M, Helfen A, Claussen J (2018). Use of multispectral optoacoustic tomography to diagnose vascular malformations. JAMA Dermatol.

